# Morphometrics and Reproductive Characteristics of the Freshwater Crab *Sartoriana spinigera* from the Habitat of Ratargul Swamp Forest, Bangladesh: An Approach to Conservation

**DOI:** 10.1155/2024/4550875

**Published:** 2024-08-21

**Authors:** Sanjida Akther, Mohammad Amzad Hossain, Debasish Pandit, Thouhidur Rahman Chowdhury, Sohel Mian

**Affiliations:** ^1^ Laboratory of Aquatic Biodiversity and Ecophysiology Department of Fish Biology and Genetics Sylhet Agricultural University, Sylhet 3100, Bangladesh; ^2^ Department of Fishery Resources Conservation and Management Khulna Agricultural University, Khulna 9100, Bangladesh

## Abstract

A total of 332 freshwater crab *Sartoriana spinigera* samples were collected for eight consecutive months from the Ratargul freshwater swamp forest, Bangladesh, to examine their general morphometrics and reproductive traits. The length-weight relationships of carapace weight with carapace length, width, and depth indicated negative allometric growth. The logarithmic relationship between carapace weight and carapace length, carapace weight and carapace width, and carapace weight and carapace depth exhibited a correlation value of 0.86, 0.79, and 0.56, respectively. Fulton's condition factor and allometric condition factor were found to be highest in March and lowest in October. The sex ratio was 1.59 : 1 for the male to female, which did not show a considerable divergence from the expected 1 : 1 ratio in the chi-square test (*P* < 0.05). In July, a maximum of mature individuals was collected and investigated. The fertilization was 1297 ± 629 ova, the ovary weight was 447.22 ± 359.39 mg, and the egg diameter was 375.15 ± 130.18 *µ*m. Histological analysis showed that the male hepatopancreas was well organized and decomposition was reported in the female during the vitellogenic stages of the ovary. Spermatogonium cells were found in the male gonad, and cells from the female gonad were in the vitellogenic mature stage. In conclusion, the research will serve as a foundation for future research work on freshwater crab species as well as the conservation and maintenance of the ecological balance of this species in the Ratargul freshwater swamp forest.

## 1. Introduction

Freshwater swamps are distinct ecosystems with particular types of vegetation and abundant invertebrate assemblages [1]. Swamp forests perform a variety of significant hydrological supporting functions, such as providing water storage space, controlling and protecting flood peaks, maintaining subsurface water levels, recharging runoff, absorbing pollutant degradation, and purifying the quality of water [2]. The Ratargul swamp forest is a distinct ecosystem in the northeast of Bangladesh with lowland vegetation, narrow depressions, and seasonal changes in water [3]. As this distinct habitat has great potential values for floral and faunal biodiversity, and other intangibles, management, and resource investigations become very important [4]. Freshwater crabs are one of the key ecologically significant invertebrate groups in tropical and subtropical water areas [5, 6]. They provide an excellent food source for many human cultures and are crucial to the food chain of the aquatic ecosystem. Crabs now contribute significantly to the fishery wealth of several countries [7]. The world's freshwater crab species are thought to serve as more than just a source of food; they are also used as fertilisers, food additives, and sources of many therapeutic elements [8]. However, little is known about the freshwater crab resources in Bangladesh, and very little research on freshwater crabs has been done so far. To form a complete inventory of species and a crab resource management program in Bangladesh, a survey is required on the range of crab resources from various regions of the country.

The freshwater crab *Sartoriana spinigera* is a substantial deep brown crab that is found moving, burrowing, and even buried in the mud soil of the littoral zone of wetlands. The environments of wetlands are the most advantageous for this crab's reproduction. *S. spinigera* was reported to be present in the Ratargul swamp forest by Islam et al. [9]. This species offers potential food value and has been reported to contain 30–59% of protein and 7–11% of lipid content [10]. However, very little is known about its reproduction, morphometrics, and sexual maturity in Bangladesh. Therefore, the current research aimed to investigate their major morphometric dynamics, sexual maturity, sex ratio, and reproductive features in the Ratargul swamp forest, Bangladesh habitat.

## 2. Materials and Methods

### 2.1. Study Area, Sample Collection, and Morphometric Measurement

**Figure 1:**
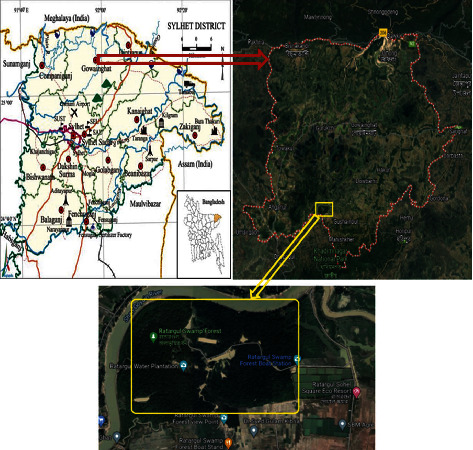
Location of the Ratargul freshwater swamp forest in Sylhet, Northeast Bangladesh (map modified from Hossain et al., [1]).

Random mature samples of *S. spinigera* were collected from Ratargul swamp forest between March and October 2022 (Figure 1). It is located in the Gowain River, Fatehpur Union, Gowainghat upazila, Sylhet, Bangladesh, and receives its flows from the Gowain River system. The forest is drowned under 6.09 to 9.10 m of water during the rainy season due to runoff from India, while the water level remains at about 3.05 m deep for the remainder of the year [1]. About 8–10 traditional bamboo traps with approximately 0.5 cm × 0.5 cm × 1.5 cm mesh sizes for adult freshwater crabs were loaded with the help of local fishermen for 8 hours in different parts of the swamps forest each month, and chopped small fish were used as bait. No additional care has been taken to attract ovigerous females. Any bycatch other than crab was immediately discarded in the water. The water quality parameters, i.e., temperature, dissolved oxygen, electric conductivity, total dissolved solids, and pH were measured on-site using a professional YSI digital multiprobe meter, Model 58, and depth was recorded for each sampling point. Just after collection, the animals were placed in large fibreglass tanks to be carried to the wet laboratory facility of Fish Biology and Genetics, Sylhet Agricultural University, Bangladesh. Major morphometric measurements, i.e., weight, carapace length, carapace width, and carapace depth, were taken using automated battery-powered vernier callipers and electric balance. The length parameters were calculated based on the description of Ashkenazi et al. [11]. The depth has been measured by putting the crabs ventrally on vernier callipers to reach the maximum depth. Details of measurement are illustrated in Supplementary Figure S1. The sex of the animals was confirmed by observing their ventral sides and the gonadal stage was confirmed by dissecting them. A part of the gonads and hepatopancreas of the mature sample was preserved in 10% neutral buffered formalin (NBF) to identify gonadal cells and liver changes in response to gonadal maturation.

### 2.2. Analysis of Length-Weight Parameters, Condition Indices, Sex Ratio, GSI, and HPI

The empirical relation of different length-weight parameters was constructed by the description of Cren 1951 [12], and *a*, *b*, and *r*^2^ parameters were obtained by building logarithmic plots between the measurement of length and weight measurement [13]. Condition factors were computed as allometric *K*_*A*_ = W/*L*^*b*^ [14], Fulton's *K*_*F*_ = 100 × (*W*/ *L*^3^), and relative *K*_*R*_ = W/(*aL*^*b*^) [12]; where *W* refers to the weight of the individual crab (g), *L* for the length of the carapace (mm), and a and *b* are the parameters obtained from the length-weight correlation. The size at first sexual maturity was calculated as log (*L*_*m*_) = ⟶0.1189 + 0.9157 × log (*L*_max_) [15]. The sex ratios were recorded in each monthly sample and compared with the chi-square test to determine the deviation from the 1 : 1 ratio. The value of the gonadosomatic index (GSI) and hepatopancreatic index (HPI) was calculated as the percentage of the ratio between the weight of the respective organ and the body weight.

### 2.3. Fecundity, Gonadal Maturation, and Histology of the Gonad and Hepatopancreas

The mature and immature phases of both male and female gonads were identified in each sampling month following the criteria of Silva et al. [16]. The female gonad is classified into four consecutive stages, i.e., stage I—immature previtellogenic phase, stage II—ripening vitellogenic phase, stage III—mature complete vitellogenic phase, and stage IV—spawned; while the male gonad is distinguished as immature stage I, maturing stage II, and mature stage III. Only mature ovaries during July were included in the estimation of fertility. Initially, three subsamples of 0.25 to 0.30 g of each ovary were separated and then diluted with Gilson fluid to disintegrate the ovum. Finally, the number of ova in all subsamples was manually counted on a stereomicroscope. Hereafter, fertilization was computed using the description of Rasheed and Mustaquim [17]. Standard histological procedure for crustaceans, followed as described by Santos et al. [18]. The method started with fixing the gonadal and liver samples in Bouin fixative. Then they were cleaned with xylene and dehydrated in graded ethanol. Paraffin-embedded blocks were sectioned and stained with hematoxylin and eosin dyes used to visualize cells under the Primo Star v3.0 microscope.

### 2.4. Data Analysis

The data were managed in Office 365 Excel tools and analyzed in SPSS v26 (IBM Corporation, Armonk, USA). The assumptions of normal distribution and homogeneity were tested by employing the Shapiro–Wilk test in SPSS. A one-way analysis of variance (ANOVA) was performed to compare the means and Tukey's HSD post hoc test was used to confirm significance at *P* < 0.05. The correlation coefficient values were calculated from Pearson's correlation and the *F* test was employed to test the significance of the regression at *P* < 0.05. The principal component analysis (PCA) between different morphometrics and water quality parameters has been constructed by using multivariate tools of software past version 4.06b and illustrated in scattered biplot.

## 3. Results

### 3.1. Water Quality Properties

The water temperature and total dissolved solids were declared to be very unstable throughout the study period, while the pH and dissolved O_2_ showed moderate variation (Table 1). The maximum water temperature was obtained in August (29.53°C) while the lowest was in March (23.33°C). The value of the TDS level reached its highest in June, while the lowest was during the prewinter seasons in October. Similarly, the pH reached its highest in April and lowest in March. The electrical conductivity was maximum in September and lowest in May.

### 3.2. Morphometrics Dynamics, Correlation, Length-Weight Parameters, and Sex Ratio

**Figure 2:**
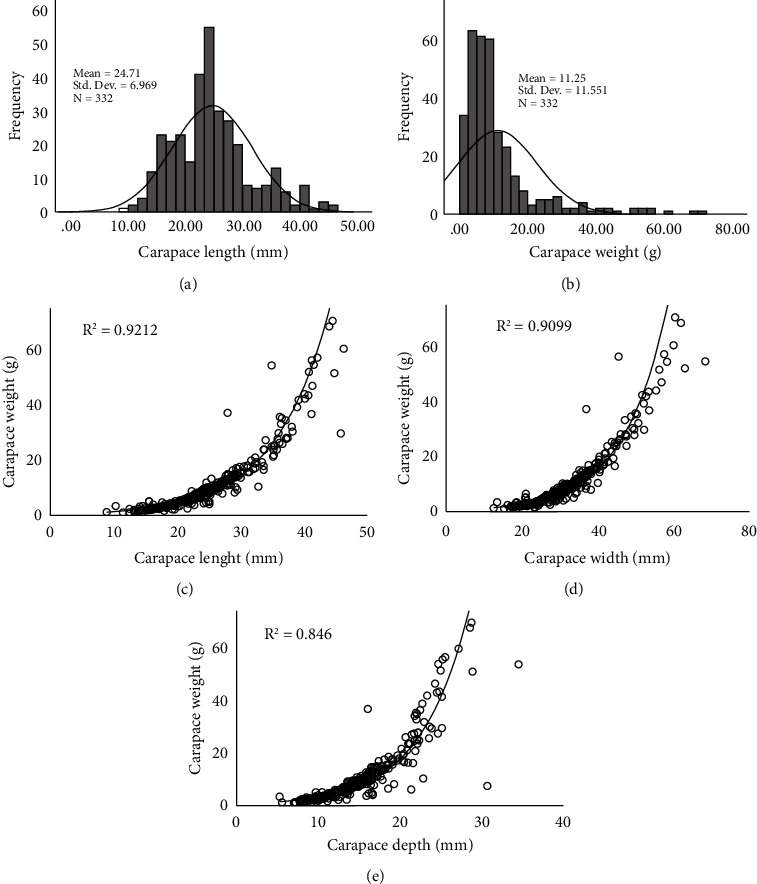
(a)–(b) Length and weight frequency distribution and (c)–(e) regression plots for the relationship between weight and length measurements.

Table 2 represents the dynamics of the main morphometrics during the sampling period. The lowest values for all morphometric parameters were obtained in May, while the highest values were obtained in July. The length and weight frequency distribution plots also revealed discrete forms of length and weight data in the studied sample (Figures 2(a) and 2(b)). The highest body weight for females, carapace length, carapace width, and carapace depth recorded as 17.69 ± 1.97 g, 30.99 ± 1.43 mm, 39.54 ± 1.78 mm, and 18.92 ± 0.95 mm, respectively, for females, and 17.16 ± 2.45 g, 27.66 ± 1.28 mm, 36.53 ± 1.85 mm, and 16.97 ± 0.96 for male in July (Table 2). Again, regression analysis of different morphometric characteristics was found to have a strong *r*^2^ value. Carapace weight and carapace length, carapace width, and carapace depth exhibited *r*^2^ values of 0.86, 0.79, and 0.56, respectively (Figures 2(c) and 2(e)).

**Figure 3:**
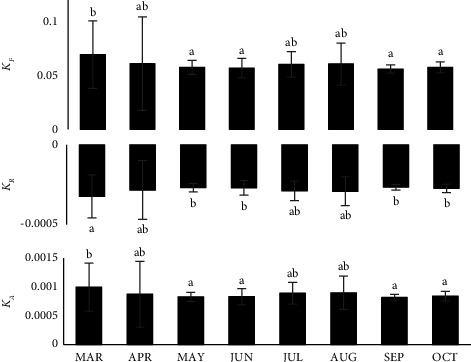
Different condition indices of *S. spinigera* in the Ratargul freshwater swamp forest (values are means ± SD; *P* < 0.05).

The *a*, *b*, and *r*^2^ parameters for different length-weight data are demonstrated in Table 3. All the weight and associated length features were calculated to be highly correlated. The growth types were negative allometries for all cases, as seen by the negative values. The length at maturity was recorded as 25.43 mm carapace length, 36.34 mm carapace width and 19.44 mm carapace depth for female and 25.21 mm carapace length, 30.39 mm carapace width, and 16.51 mm carapace depth for male. The *b* values were below 3 for all cases as well. Fulton's condition factor *F*_*k*_ and the allometric condition factor *K*_*A*_ followed the same pattern of variation throughout the sampling period, occupying a peak in March, while the relative condition factor *K*_*R*_ represents several peaks that are opposite to the above condition factors (Figure 3).

Monthly changes in the sex ratio of collected samples are shown in Table 4. A total of 204 male and 128 female crabs had been spotted during the study period. The *χ*^2^ test at *P* < 0.05 revealed that the male and female ratio of the crab samples collected varied significantly from the 1 : 1 ratio. A moderately male-dominant population had been estimated in most of the sampling periods.

**Figure 4:**
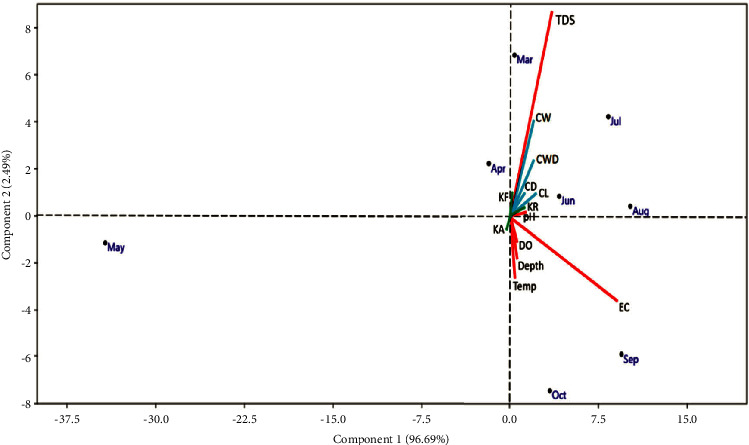
PCA analysis of morphometrics with water quality parameters (CW-carapace weight, CL-carapace length, CWD-carapace width, CD-carapace depth, DO-dissolved O_2_, total dissolved solids TDS, EC-electric conductivity).

The PCA plots (Figure 4) showed a clear formation of TDS, SW, CW, CD, CL, *K*_*F*_, *K*_*R*_, pH group, and *K*_*A*_, DO, depth, temperature, and electric conductivity group. All variables showed moderately strong influence with different monthly slots. The PCA between morphometrics and water quality in different months is explained as 96.69% and 2.49% of the total variance.

The water parameters showed a slight to moderate correlation with most morphometrics in March, July, June, August, October, and September. However, DO, depth, temperature, and EC were found to have a weak correlation with allometric condition factors in May, October, and September.

### 3.3. Hepatopancreatic Index (HPI) and Gonadosomatic Index (GSI)

**Figure 5:**
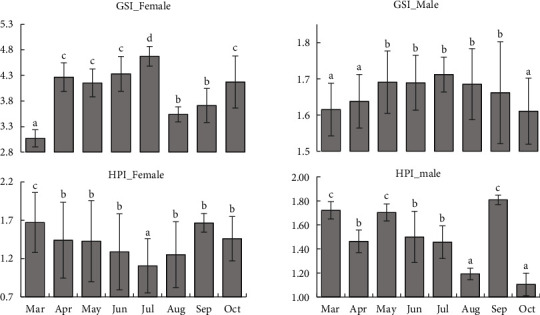
Hepatopancreatic index (HPI) and gonadosomatic index (GSI) in S. spinigera (values are means ± SD); different superscripts indicated significance differences at *P* < 0.05.

Female individuals exhibited the highest HPI during March and the lowest during July (Figure 5). In contrast, the peak of GSI in females was reported in July and the lowest in March. On the other hand, the HPI values in male crabs showed numerous peaks in March, May, and September, with the lowest value in October. The GSI value of males has also been reported to show several peaks between May and September.

### 3.4. Gonadal Maturation Phases, Histology, and Fecundity

**Figure 6:**
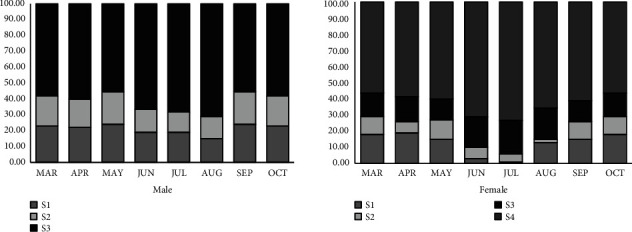
Monthly dynamics of gonadal maturation stages.

Different phases of mature and immature gonadal stages have been reported for the entire studied period (Figures 6(a) and 6(b)). The highest mature ovaries occupying stage 4 and testes in stage 3 were spotted in June, July, and August. Crabs having matured or final stages of ovum were only used for fecundity estimation. The fecundity of the female crabs collected during July was accounted as 1297 ± 629 ova with an average ovarian weight of 447 ± 359.39 mg and an ovarian diameter of 375.15 ± 130.18 *μ*m.

**Figure 7:**
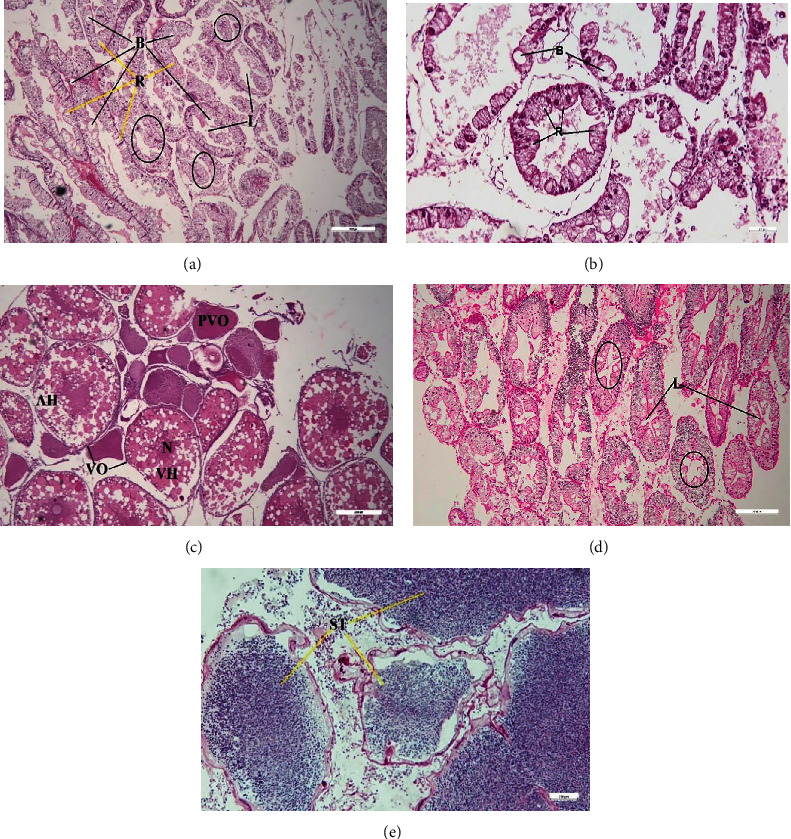
Histology of liver and gonads in mature crabs collected in July 2022 ((a)–(c) female, (d)–(e) male) (L-hepatic lumina, black circle-lumen structure in male and female hepatopancreas, B-B cell, R-R cell, ST-spermatids, N-nucleus, AH-animals pole, VH-vegetal pole, PVO-previtellogenic oocyte, VO-vitellogenic oocytes).

The mature female ovaries were characterized by the presence of vitellogenic and previtellogenic oocytes with well-pronounced nuclei, animal poles, and vegetal poles (Figure 7(b)). Again, the histology of the testes revealed the presence of mature spermatid cells (Figure 7(e)). Increased liver B cells and disintegration of lumen structure in mature stages of female hepatopancreases were recognizable (Figures 7(a) and 7(b)) in liver tissue, while there was no such sign in the male liver (Figure 7(d)).

## 4. Discussion

The isometric value of *b* (>3) seems to be common in crustaceans. A *b* value of 2.931 in blue crab *Callinectes sapidus* [19], 2.71 in brown crab *Charybdis callianassa* [20], and 2.91 in *Potamon algeriense* [21] had already been documented. The current research also demonstrated a value of *b* between 2.60 and 2.89 for different length-weight relationships. Again, several studies also well reported a strong correlation between the length and weight parameters of crustaceans. An *r*^2^ value of 0.97 in mud crab *Scylla olivacea* [22], 0.906 in *Callinectes sapidus* [19], and 0.993 in *Potamon algeriense* [21] were noted as well. The length at sexual maturity was reported to be between 27 and 32 mm for both sexes in the freshwater crab *Potamon algeriense* [21], and 108–130 mm for female brown crab *Cancer pagurus* [23, 24]. However, the size at maturity differs between species and is highly dependent on the methodology or model applied to the calculation [23, 25]. A value of *r*^2^ 0.81 has been documented for this species in East Kolkata Wetland, India, with a size range of 32–37 mm carapace length [26]. Therefore, the findings of current research follow similar trends in different length-weight parameters.

The DO level above 5 mg L^−1^ and pH ranged from 5 to 10 deemed reasonable to favour the growth of the majority of aquatic macro-fauna [27, 28]. The measurement of turbidity and water temperature in the current research was ideal for the growth of tropical aquatic organisms [27, 29]. A very similar form of water quality parameters has already been documented in the Ratargul Swamp Forest and other freshwater habitats in Bangladesh [1, 30, 31]. Thermal alterations are due to periodic fluctuations in temperature. The highest turbidity in July was due to heavy rainfall and the mass siltation of anthropogenic runoff. Additionally, the escalating trend of O_2_ in the rainy season suggested better saturation associated with increased water circulation. The water characteristics registered in this study were found to be within the optimal range for aquatic organisms' growth and reproduction [27, 32]. Nur Syafaat et al. [33] reported that the physiology of mud crab *Scylla paramamosain* is triggered in large part by water quality parameters which in turn results in changes in growth and morphometrics. Hossain et al. [1] recorded a moderate to strong correlation between different parameters of water quality and the growth of freshwater mussels. The sheltering environment, water quality, and food availability are key parameters in determining the morphometric features of crabs [34]. The correlation between morphometrics and water quality characteristics in the current research also remained supportive of previous reports.

Male dominance in different crab populations is well-established in several studies [21, 35]. A female-to-male sex ratio distorted towards 1 : 0.3 had been observed in freshwater crab *Trichodactylus fluviatilis* [36]. A distinctive population of males (94%) and females (74%) has already been reported among three sympatric *Scylla* species has already been reported [37]. It is already been reported that male crabs have a higher tendency to disperse more in comparison to females [38]. A moderately higher abundance of females in August, September, and October might be due to environmental factors. Elevated rainfall therefore increasing water depth in sampling sites makes the crabs dispersed more widely. The current research aligned with the different reports of previous observations. Low fertilization seems very common in freshwater crabs. Abit et al. [39] reported fecundity ranged from 26 to 81 eggs, with an ovum diameter of 3.7 to 4.2 mm in Purple crab *Isolapotamon bauense* from Sarawak, Malaysia, while 340 to 800 ova were accounted for *Potamon mesopotamicum* from Iraq [40], and 533–1306 ova for *Paratelphusa spinigera* in India [41]. Therefore, *S. spinigera* could be treated as a slightly higher-fecundity freshwater crustacean like *Paratelphusa spinigera* in India [41].

The value of GSI seemed to increase during the onset of the breeding season among aquatic animals [25]. The value of GSI ranged from 3 to 5 for both sexes in the crab *Sesarma intermedia* [42]. Higher GSI values were reported in *Scylla olivacea* before the breeding season [25]. The current research also showed a strong increase in the value of GSI in response to the breeding season. The value of HPI in females tends to be inversely related to GSI, which is also reported in several aquatic animals [43]. This is due to the release of vitellogenin from hepatic tissue to the ovary [44], which is also evident in the intense degeneration of the hepatic lumen in female crabs. Aaqillah-Amr et al. [45] stated that oocyte development was strongly related to GSI in *S. serrata,* and increased B cell and R cells were reported in the hepatopancreatic tubules as the ovarian development process progressed. Mekuleyi and Fakoya [46] also described hepatopancreas changes during the onset of advanced ovarian development in C. *pallidus*. However, no such trend was described in HPI of male crabs, and crabs are reported to have mature gonadal cells throughout the year [47]. The higher GSI value for both sexes reported in June and July suggesting breeding peak for this species at studied area. Also, water quality properties in this month favour the availability of more food and suitable parameters for breeding.

In conclusion, the length-weight relationship showed negative allometric growth and the sex ratio of *S. spinigera* did not show a considerable deviation in the study area. The calculated length-weight relationship and different condition factors in the current research will be valuable data sources for the establishment of a monitoring and management system of these species in different freshwater ecosystems. This research may also help to conserve *S. spinigera* species as an unconventional fisheries resource by providing key data on their morphometrics and reproduction.

## Figures and Tables

**Table 1:** Major water quality parameters at the study site.

	Temperature (°C)	Dissolved O_2_ (mg L^−1^)	Electric conductivity (*µ*S cm^−1^)	Total dissolved solids (mg L^−1^)	pH	Depth (m)
March	23.23 ± 0.33^a^	4.46 ± 0.15^a^	62.84 ± 1.30^a^	32.17 ± 0.44^c^	6.24 ± 0.09^ab^	1.64 ± 0.09^a^
April	28.40 ± 0.37^bc^	4.76 ± 0.06^ab^	62.32 ± 1.22^a^	30.41 ± 1.33^bc^	6.66 ± 0.20^ab^	1.70 ± 0.06^a^
May	27.91 ± 0.18^b^	5.15 ± 0.21^bc^	58.13 ± 2.53^a^	24.88 ± 1.57^ab^	6.60 ± 0.22^ab^	2.69 ± 0.13^b^
June	27.67 ± 0.42^b^	5.48 ± 0.16^bc^	67.94 ± 0.64^b^	29.68 ± 0.32^bc^	7.15 ± 0.27^b^	3.17 ± 0.02^b^
July	29.38 ± 0.22^c^	5.46 ± 0.12^bc^	69.96 ± 0.58^bc^	32.14 ± 0.53^c^	6.88 ± 0.15^b^	3.06 ± 0.28^b^
August	29.65 ± 0.31^c^	5.48 ± 0.22^bc^	72.93 ± 0.47^bc^	29.49 ± 1.22^bc^	6.67 ± 0.14^ab^	3.31 ± 0.08^b^
September	29.53 ± 0.36^c^	5.62 ± 0.09^bc^	75.19 ± 0.83^c^	25.76 ± 2.88^abc^	6.71 ± 0.04^ab^	3.04 ± 0.31^b^
October	27.28 ± 0.28^b^	5.23 ± 0.17^bc^	70.17 ± 0.35^bc^	20.95 ± 1.49^a^	5.92 ± 0.28^a^	3.19 ± 0.12^b^

The column with different superscripts indicates significant differences at *P* < 0.05; values are means ± SD.

**Table 2:** Different growth morphometrics of *S. spinigera.*

Months	Weight (g)	Carapace length (mm)	Carapace width (mm)	Carapace depth (mm)
*Male*				
March	14.99 ± 3.86^ab^	23.46 ± 1.80^ab^	31.49 ± 2.63^ab^	14.18 ± 1.16^ab^
April	9.69 ± 2.27^ab^	22.65 ± 1.56^ab^	29.46 ± 2.04^ab^	13.82 ± 0.94^ab^
May	5.59 ± 0.45^a^	20.76 ± 0.66^a^	27.10 ± 0.88^a^	12.33 ± 0.42^a^
June	10.07 ± 1.90^ab^	23.55 ± 1.22^ab^	30.71 ± 1.71^ab^	14.37 ± 0.73^ab^
July	17.16 ± 2.45^b^	27.66 ± 1.28^b^	36.53 ± 1.85^b^	16.97 ± 0.96^b^
August	10.49 ± 1.78^ab^	24.35 ± 0.75^ab^	31.64 ± 1.02^ab^	15.57 ± 1.01^ab^
September	7.78 ± 0.95^ab^	23.24 ± 0.89^ab^	30.14 ± 1.20^ab^	13.79 ± 0.61^ab^
October	8.81 ± 2.4^1ab^	22.66 ± 1.56^ab^	29.27 ± 2.25^ab^	13.25 ± 0.94^ab^

*Female*				
March	11.83 ± 2.87^ab^	26.68 ± 3.06^ab^	33.79 ± 3.17^ab^	15.87 ± 1.52^a^
April	7.12 ± 0.84^a^	24.07 ± 1.08^a^	30.82 ± 1.27^a^	15.22 ± 0.96^a^
May	7.56 ± 0.53^a^	23.98 ± 0.68^a^	31.03 ± 1.01^a^	14.97 ± 0.57^a^
June	12.10 ± 1.89^ab^	27.51 ± 1.24^ab^	35.03 ± 1.66^ab^	17.43 ± 0.85^a^
July	12.74 ± 2.68^ab^	26.68 ± 1.65^ab^	34.10 ± 2.04^ab^	16.22 ± 1.09^a^
August	17.69 ± 1.97^b^	30.99 ± 1.43^b^	39.54 ± 1.78^b^	18.92 ± 0.95^a^
September	10.53 ± 1.21^ab^	26.04 ± 1.01^ab^	33.13 ± 1.19^ab^	15.47 ± 0.61^a^
October	11.28 ± 1.25^ab^	26.03 ± 1.07^ab^	33.30 ± 1.37^ab^	15.81 ± 0.69^a^

The column with different superscripts indicates significant differences at *P* < 0.05; values are means ± SD.

**Table 3:** *a*, *b*, *r*^2^, and *L*_*m*_ parameters of *S. spinigera.*

Equation	*a*	*b*	*r* ^2^	95% confidence interval		*L*_*m*_ (mm)		GT
				*a*	*b*	Female	Male	
*CW*=*a* × *CL*^*b*^	−3.07	2.89	0.86	−3.16–3.06	2.86–2.93	25.43	25.21	A−
*CW*=*a* × *CWD*^*b*^	−3.32	2.81	0.79	−3.48–3.47	2.93–2.94	36.34	30.39	A−
*CW*=*a* × *CD*^*b*^	−2.12	2.60	0.56	−2.30–2.23	2.70–2.75	19.44	16.51	A−

*N*-sample size, *r*^2^-correlation coefficient, CW-carapace weight, CL-carapace length, CWD-carapace width, CD-carapace depth, Lm length at maturity, GT-growth type, *A*-refers to negative allometries.

**Table 4:** Sex ratio of *S. spinigera* from the Ratargul freshwater swamp forest, Bangladesh.

Months	Total	Male		Female		*M* : *F*
		Number	%	Number	%	
March	41	32	78.04	9	21.95	3.56 : 1
April	41	30	73.17	11	26.83	2.73 : 1
May	39	28	71.79	11	28.21	2.55 : 1
June	45	29	64.44	16	35.56	1.81 : 1
July	50	32	64.00	18	36.00	1.78 : 1
August	36	18	50.00	18	50.00	1.00 : 1
September	39	17	43.58	22	56.41	0.77 : 1
October	41	18	43.90	23	56.10	0.78 : 1
Total	332	204		128		1.59 : 1

## Data Availability

Data that support the current findings will be available from the corresponding author on reasonable request.

## Supplementary Materials

Supplementary Materials Figure S1: Morphometric measurements of the freshwater crab *Sartoriana spinigera*.
