# Supplementary figures and images for: Morphometrics and Reproductive Characteristics of the Freshwater Crab Sartoriana spinigera from the Habitat of Ratargul Swamp Forest, Bangladesh: An Approach to Conservation
Source: Scientifica (Cairo). 2024 Aug 21;2024:4550875. doi: 10.1155/2024/4550875 (PMC11357817; doi:10.1155/2024/4550875)

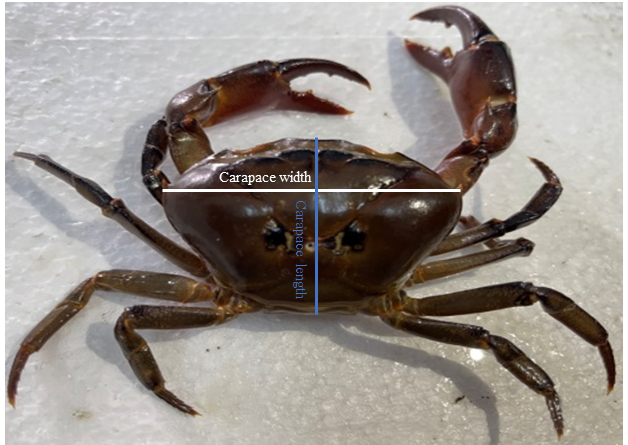


Figure S1 Morphometric measurements of the freshwater crab *Sartoriana spinigera.*

Supplement: Supplementary Materials — Figure S1: Morphometric measurements of the freshwater crab Sartoriana spinigera. [file 4550875.f1.docx]
